# Information Technology Use and Cyberbullying Behavior in South Thailand: A Test of the Goldilocks Hypothesis

**DOI:** 10.3390/ijerph17197122

**Published:** 2020-09-29

**Authors:** Ruthaychonnee Sittichai, Peter K. Smith

**Affiliations:** 1 Information Management Program, Kids and Youth Development Research Unit, Research Center for Educational Innovations and Teaching and Learning Excellence, Faculty of Humanities and Social Sciences, Prince of Songkla University, Pattani 94000, Thailand; 2 Department of Psychology, Goldsmiths University of London, London SE14 6NW, UK; p.smith@gold.ac.uk

**Keywords:** ICT use, cybervictim, curvilinear trend, Thailand, internet, Goldilocks Hypothesis, cyberbullying, victimization

## Abstract

There has been concern about the effects of high levels of internet use on the mental well-being of young people. This has generally been phrased in terms of a displacement hypothesis, that the extent of internet use and mental well-being are directly proportional. This linear model has been contrasted with a Goldilocks Hypothesis, proposed by Przybylski and Weinstein. This supposes that moderate levels of internet use may be the least harmful, conforming to a curvilinear relationship. Here these hypotheses were tested on a sample of 1140 adolescents (42% boys, 58% girls) aged 12–18 years, in 12 schools from Southern Thailand. We first report levels of internet use, and of cybervictimization, taken as one important aspect of mental well-being. We then assess the relationship of four factors of internet use (frequency, time spent, number of places accessed, number of activities) with (a) being a victim of cyberbullying, and (b) being a frequent victim; taking these as indicators of mental well-being. For (a) there was limited evidence of a Goldilocks effect on two out of four measures. For (b) the evidence did support a Goldilocks effect for all four measures, but these were under-powered analyses and the findings did not reach statistical significance. If substantiated on larger samples, a curvilinear relationship between aspects of internet use and cyberbullying would suggest a ‘safe zone’ for adolescent internet use, bringing its benefits while minimizing risks of cyberbullying. In the future, similar research should use larger sample sizes or longitudinal measures when exploring nonlinear trends and include other aspects of mental well-being.

## 1. Introduction

An accurate measure of the connection between internet use and cybervictimhood could provide better data for managing cyberbullying and helping cybervictims. This study considers whether, besides any linear relationship, a curvilinear relationship may also be found in the connection between internet use and cybervictimization of adolescents.

The internet and related technologies have become an important aspect of everyday life, especially for young people. They are widely used for communication information, education, and entertainment. Technological advances offer young people a plethora of options for social connections such as web blogs, websites for sharing pictures, songs, videos, chat boards, emails, and quick messages. Many websites cater specifically to social networking users, such as Google Group, Facebook, Twitter, MySpace, and YouTube. Social networking has greatly influenced the lives of teenagers [1].

Internationally, mobile, social media and internet use has been on the rise, and this is the case in Thailand, with a majority of the population being internet users [2]. By 2013, when our data were obtained, average time on the internet was already 16 h per week [3]. Most youth connect to the internet through a mobile device, primarily for entertainment followed by educational purposes; and a very popular social media site is Facebook [4,5]. Furthermore, mobile phones double as status symbols [5,6]. Pongput [7] reported that experiences on the internet in Thailand are generally positive, because it provides opportunities for developing knowledge, friendships, and personal expression use. Unfortunately, internet usage has also opened the door for more negative aspects of internet connectivity, such as cyberbullying.

### 1.1. Possible Negative Effects of Adolescent Internet Use

There can be risks with internet use, such as exploitation, grooming, and cyberbullying [8]. These risks are thought to relate to the frequency of internet use. Many studies have correlated indices of internet use with measures of mental well-being, suggesting negative relationships, and indeed excessive internet use, or internet addiction, has been seen as a new kind of clinical phenomenon [9].

In a meta-analysis of 61 studies of time spent on social networking sites, Huang [1] reported an overall correlation with mental well-being of *r* = −0.07, statistically significant although of small effect size. Similar negative relationships have been reported in recent large and nationally representative surveys in the USA and UK. In the USA, Twenge et al. [10] reported that adolescents who spent more time on new media were more likely to report mental health issues such as depression and suicide-related behaviors. In the UK, Kelly et al. [11] found that greater social media use in 13–18 year old related to online harassment, poor sleep, low self-esteem, and poor body image. In a different cohort study, Khouja et al. [12] found significant although small relationships between time 16 year olds spent on a computer, and anxiety and depression at 18 years. In general, there appear to be negative relationships between ICT (information and communications technology) use and measures of well-being, but often of small effect size [13]. However, such correlations appear consistent and rather more substantial when measures of very high level or excessive internet use are assessed [14,15].

Studies of ICT use have generally focused on time spent online, or frequency of internet use. However two other aspects, perhaps particularly relevant for adolescents, are the number of different places where the internet is accessed; and the number of different activities that the internet is used for. We included all these measures in our data collection, giving four measures of ICT use in total.

### 1.2. Two Contrasting Hypotheses

Przybylski and Weinstein [16], contrasted two hypotheses regarding the possible negative effects of greater internet use for mental well-being. The first is the displacement hypothesis. This is characterized as a traditional view, rooted in postulated negative effects of television viewing [17], as displacing or supplanting alternative activities such as socializing with family or friends, or engaging in more active leisure pursuits. This hypothesis has been applied to digital-screen use and the internet, both in terms of displacing more traditional media activities, and other socializing and leisure activities [18,19,20]. This hypothesis supposes a linear relationship between increasing digital use and harm, due to the loss of normal socializing experiences, the risks of using the internet, and internet addiction. Many studies (cited above) have reported linear trends which tend to support this point of view.

However, an exclusive focus on linear trends may obscure the benefits of moderate internet use, and underestimate the negative effects of excessive internet use. Indeed, it may lead to the findings of negative relationships of small magnitude often reported, even when findings for very high level or excessive internet use are much more substantial. Przybylski and Weinstein [16] contrasted the displacement hypothesis with what they termed a Goldilocks Hypothesis. This is named after the Goldilocks children’s story, where Goldilocks (a young girl) tastes three bowls of porridge. One is ‘too hot’, one is ‘too cold’, but the third is ‘just right’. The story serves to illustrate that moderate internet use may be the most optimal, neither too high nor too low to cause harm. Przybylski and Weinstein [16] tested this using four measures of digital-screen time, and a mental well-being scale, on a large sample of 15 year olds in the U.K. On all four measures their Goldilocks Hypothesis was supported, with optimal mental well-being reported by those with a low, but not the lowest, levels of digital-screen engagement. They also contrasted weekday and weekend use, but obtained similar curvilinear trends in both cases. Previous research on internet use and mental well-being reported linear trends in the data. However, the Przybylski and Weinstein [16] report establishes the importance of considering curvilinear trends.

Twenge [21] summarized data from the USA and the UK, suggesting curvilinear trends between digital media use and mental well-being: risk is slightly higher with very low users, least for low-to-moderate-users, and higher for intensive or excessive users. However, in one test of the Goldilocks hypothesis, on a U.S. sample of adolescents, there was little evidence of effects of IT (information technology) use on mental health, or of quadratic effects [22]. Viner et al. [23] with a sample of 13–16 year olds from England, did not explicitly test for the Goldilocks effect, but their data do show such an effect on the General Health Questionnaire (this measure being better for social media use ‘every couple of days’ compared to either ‘weekly or less’ or to ‘once daily’ or more frequently). Their data also show a Goldilocks effect for having experienced any cyberbullying (here, this risk being least for social media use ‘once daily’, compared to either ‘every couple of days’ or ‘less’, or to ‘2–3 times daily’ or more frequently). In another study from England, with 18 year olds, Mars et al. [24] found mainly linear trends linking time spent online with depression, and nonsignificant for anxiety and self-harm, although curvilinear trends are suggested in their data for females.

### 1.3. Being a Cyber Victim as a Measure of Mental Well-Being

Bullying is usually defined as being an aggressive, intentional act that is carried out by a group or an individual, repeatedly and over time, against a victim who cannot easily defend him or herself [25]. It is based on an imbalance of power and can be defined as a systematic abuse of power [26]. Traditional or offline bullying takes several forms, but in the last decade especially, cyberbullying has emerged through the use of modern communication technologies [27].

Cyberbullying is typically defined as aggression that is carried out intentionally and repeatedly via mobile phones and the internet, against a person who is not able to easily defend him/herself (the victim) [27,28,29]. Cyberbullying can be perpetuated via phone calls, text messaging, instant messaging, e-mail, chat rooms, or on social networking sites such as Facebook and Twitter. Being a victim of cyberbullying has been shown by many studies to predict adverse mental health outcomes, such as depression and suicidal ideation [27,30]. The risk of being a cybervictim can be taken as one indicator of mental well-being.

A number of studies internationally have shown that the extent of internet use is one predictor of being a cybervictim; in the UK [29], the USA [31], Belgium [32], Switzerland [33], and mainland China [34]. However, all of these studies essentially obtained a linear correlation, or only compared high and low internet use groups. Sampasa-Kanyinga and Hamilton [35] reported a dose-response relationship between social networking site use, and risk of cybervictimization. Viner et al. [23] reported a curvilinear effect, but their analysis and discussion was phrased in terms of the risks of ‘very frequent media use’. Here we carry out an explicit analysis to examine whether the Goldilocks hypothesis explains the relationship between internet use and being a cybervictim using data from Southern Thailand.

### 1.4. Studies of Cyberbullying in Thailand

Although there have been many studies of cyberbullying internationally, there are relatively few in Thailand. Sittichai and Smith [36] reported a review of studies on bullying and cyberbullying in South-East Asian countries, including Thailand, before and up to 2014. Since 2014 studies of cyberbullying in Thailand among school students include [4,37,38], and [39,40] among university students. These studies have documented a variety of cyberbullying behaviors, and an appreciable incidence of the phenomenon, with actual incidence levels depending on the definitions and criteria. However, they have not focused on the relationship between internet use and being a cybervictim.

### 1.5. Objectives

Our aims were initially, to document levels of internet use amongst young people in Southern Thailand, and levels of cybervictimization. Then, we aimed to examine the relationship between cyberbullying victimization and four measures of internet use: frequency of internet use, hours of internet use per week, number of locations of internet use, and number of internet-based activities. Specifically, we tested for linear and curvilinear trends, to assess whether the displacement hypothesis or Goldilocks hypothesis better explained the relationships found. Establishing an accurate relationship could help adolescents and their caretakers mitigate the risks of cyberbullying without forfeiting the benefits of internet use. This study is the first of its kind in Thailand, and was carried out in 2013; internet use was already very prevalent then ([6,7]; and our findings, Table 1). We took the opportunity to examine this data retrospectively to see if curvilinear trends could be detected.

## 2. Materials and Methods

### 2.1. Research Design

A cross-sectional survey was conducted during March 2013 with students aged 12–18 years in Pattani, Yala, and Narathiwat provinces in southern Thailand, targeting 400 students from each province. They were drawn from 12 schools; 4 schools for each province, each comprising 2 religious schools and 2 general public schools.

### 2.2. Sample

The initial sample of 1200 fell to 1183 who responded; after excluding 19 participants with missing data, and 24 pupils who said they did not use the internet, this fell to a final total of 1140 adolescents used in the current analyses. Of these, 42% were male and 58% female. Most, 67.5%, were Islamic, with 31.7% Buddhist, and a few Christian or no response. The mean age was 15.35 years, *sd* = 1.59, range 12–18 years.

### 2.3. Data Collection

Participating adolescents were informed about the research and taking part was voluntary. The informed consent procedure was approved by Prince of Songkla University, Ethical Committee (PSU 001.02/665). If an adolescent signed the consent form, he or she was given a questionnaire on experiences with internet use, and also experiences of bullying and cyberbullying, based on one used previously in England [29] and available at the time. Following demographic information, the questionnaire asked about four measures of internet use. This was followed by definitions of bullying and cyberbullying, and adolescents were asked to respond to a question about being cyberbullied, with two measures taken from this. Data collection was done by the first author with the help of experienced assistant researchers; participants were given 40 min to an hour to complete the questionnaire.

### 2.4. Internet Use

For the four measures of internet use we examined the distribution of responses across the response options, shown in Table 1. We collapsed some categories to get an adequate distribution (at least 10% of responses in each category). We recoded as follows:

- How often do you use the internet? Participants chose 1 out of 5 options. Recoded by collapsing once a month and once a week, so a 4-point scale: once a month or once a week (*n* = 210; 18.4%), several times a week (*n* = 321; 28.2%), once a day (*n* = 265; 23.2%), and several times a day (*n* = 344; 30.2%).
- How long do you spend on internet per week? Participants chose 1 out of 5 options. Recoded by collapsing 15–20 h and 20 or more hours, so a 4-point scale: 0–5 h (*n* = 594; 52.2%), 5–10 h (*n* = 297; 26.1%), 10–15 h (*n* = 118; 10.4%), 15 or more hours (*n* = 130; 11.4%).
- Where are you most likely to use the internet?—this had 8 places; participants chose all that applied to them. We scored the number of places used, onto a 4-point scale: 1 place (*n* = 199; 17.5%), 2 places (*n* = 534; 46.9%), 3 places (*n* = 242; 21.3%), and 4 + places (*n* = 163; 14.3%).
- What activities do you use the internet for?—this had 10 activities; participants chose all that applied to them. We scored the number of activities mentioned, onto a 4-point scale: 1–2 activities (*n* = 327; 28.8%), 3–4 activities (*n* = 352; 31.0%), 5–6 activities (*n* = 248; 21.9%), and 7 + activities (*n* = 208; 18.3%).

Cyber victimization: definitions of bullying and cyberbullying were given. Bullying was first defined as ‘an action carried out by a group or individual that is repeated over time in order to hurt, threaten, or frighten an individual with the intention to cause distress. It is different from other aggressive behavior because it involves an imbalance of power which leaves the victim defenceless’; then cyberbullying was defined as ‘a new form of bullying which involves the use of e-mail, instant messaging, chat rooms, websites, mobile phones, or other forms of information technology to deliberately harass, threaten, or intimidate someone. Cyberbullying can include such acts as making threats, sending personal, racial or ethnic insults, or repeatedly victimizing someone through electronic devices’. It should be noted here that although repetition is a key aspect of traditional or offline bullying, it is not seen as so crucial in cyberbullying, since a perpetrator’s single action is typically passed on or liked by others, and this can be anticipated by both the perpetrator and the victim [27,41].

**Figure 1:**
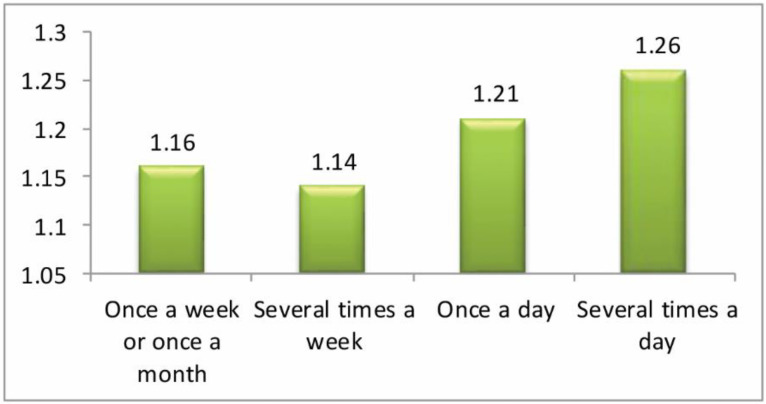
The risk of being a cybervictim (4-point scale), by the frequency of using the internet for the adolescents who used the internet (*n* = 1140).

**Figure 2:**
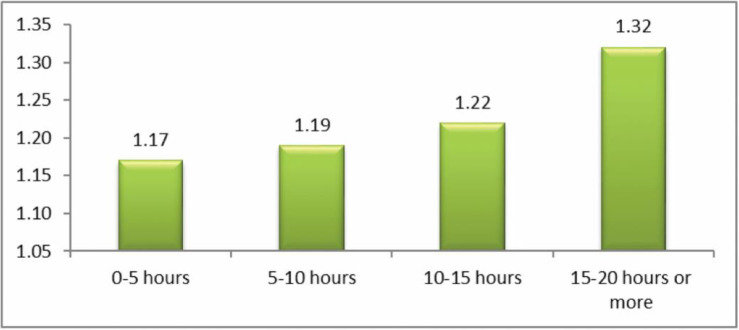
The risk of being a cybervictim (4-point scale), by time spent per week on the internet for the adolescents who used the internet (*n* = 1140).

**Figure 3:**
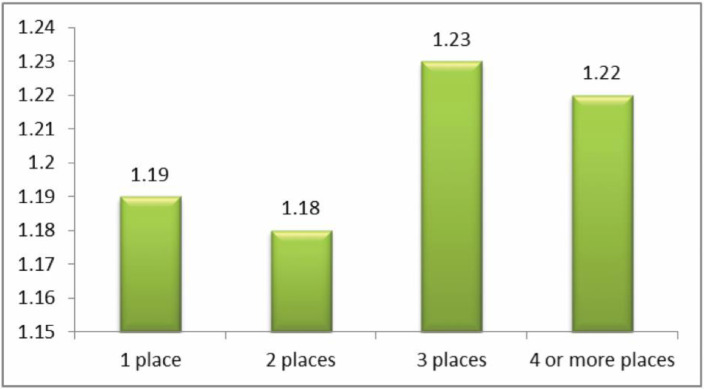
The risk of being a cybervictim (4-point scale), by numbers of sites used for internet access for the adolescents who used the internet (*n* = 1140).

**Figure 4:**
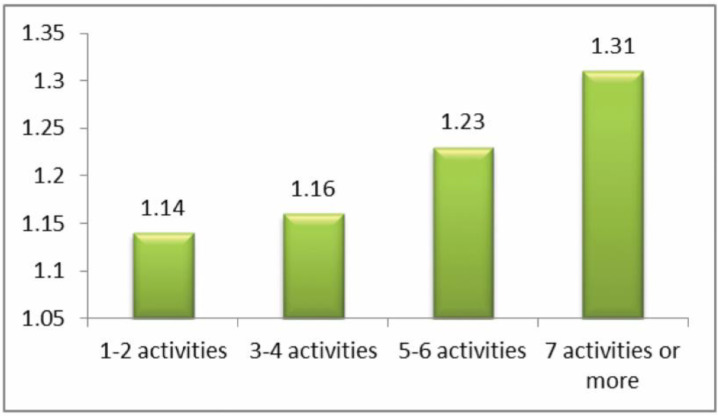
The risk of being a cybervictim (4-point scale), by number of activities on the internet for the adolescents who used the internet (*n* = 1140).

**Figure 5:**
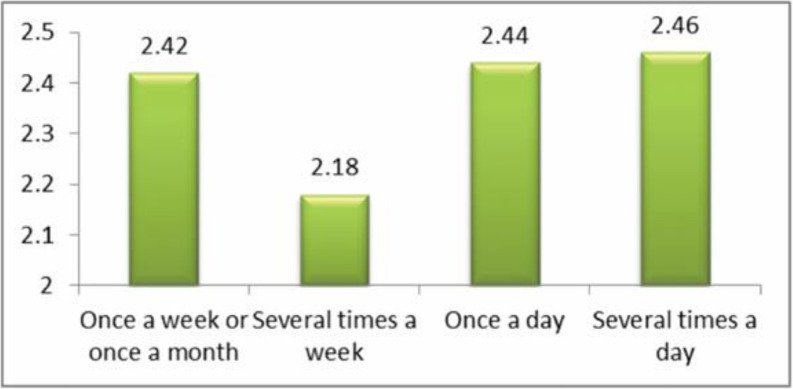
The risk of being a cybervictim (4-point scale), by the frequency of using the internet for the adolescents who were cyberbullied (*n* = 163).

The question analyzed here was: ‘how often have you been cyberbullied at school in the past couple of months?’ This had 5 response options: I have not been cyberbullied (*n* = 964, 84.6%); only once or twice a month (*n* = 126, 11.1%); 2 or 3 times a month (*n* = 19, 1.7%); about once a week (*n* = 11, 1.0%); and several times a week (*n* = 7, 0.6%). There was no response from 13 participants (1.1%). From these we took a frequency measure of being a cybervictim: scores were from 1–4 on the scale only once or twice a month (1), 2 or 3 times a month (2), about once a week (3), and several times a week (4). We first examined the risk of being a cybervictim out of the total sample of *n* = 1140. Mean frequency scores of being a cybervictim are shown in Figure 1, Figure 2, Figure 3 and Figure 4. We then examined the risk of frequent cyber victimisation for the sample of *n* = 163 who had ever been a cybervictim. Mean frequency scores of being a cybervictim for this smaller sample are shown in Figure 2, Figure 3, Figure 4 and Figure 5.

### 2.5. Data Analysis and Statistical Power

Data management and analysis were performed by SPSS Version 23 (IBM, Amonk, NY, USA). We measured the relationship between the four measures of internet use and the two measures of cybervictimization. We tested for significance of linear and curvilinear trends with both linear regression and quadratic analysis. We used *t*-tests to further examine differences between means. We calculated Cohen’s *d* for effect sizes where appropriate, to indicate the standardized difference between two means. Przybylski and Weinstein [16] found that the average effect size (Cohen’s *d*) for engagement in excess of the inflection points was −0.18. Regarding statistical power for our study, with an alpha error of 5% and a power of 80%, we would need an *n* of 394 to detect statistically significant differences with *d* = 0.20, and an *n* of 1571 at *d* = 0.10. Our full sample of 1140 is therefore satisfactory, although our subsample of 163 is under-powered.

## 3. Results

### 3.1. Internet Use

We found that adolescents often used the internet to do homework or assignments from schools, and commonly used it to download songs, movies, and programs, to play games, to send and receive emails, to chat, to surf the internet, to use other social media such as MSN or Yahoo, and to use Skype (Table 1). As expected, the great majority of the adolescents (88%) had their own private mobile phones, and about half (51%) had smartphones. Either on their smartphones, or in an internet café or at home or school, all had experience on the internet. This varied a lot in extent but typically totaled several hours each week. Facebook was the most popular activity.

The response options for the four measures of internet use are shown in Table 1, with numbers and percentages of responses for each category. The recoded categories were analyzed in relation to the two measures of cybervictimization.

### 3.2. Internet Use and Risk of Being a Cyber Victim

A comparison of the risk of ever being a cybervictim in the last couple of months, against how often a student uses the internet, is shown in Figure 1. The linear effect is significant, *b* = 0.393, *R-square* = 0.717, *p* < 0.001; the quadratic effect is also significant, *b* = 0.939, *R-square* = 0.798, *p* < 0.001. There is a curvilinear trend, with the least risk being at several times a week, rather than either once a week/once a month, or once a day or several times a day. *T* tests between several times a week, and once a week/once a month, *t* = −0.427, or once a day, *t* = −1.587, are not significant, but with several times a day, *t* = −2.662, is significant at *p* < 0.05. This provides some support for a Goldilocks effect, but with Cohen’s *d* = 0.211 this is a very small effect size.

A similar comparison for how long do you spend on the internet per week, is shown in Figure 2. Here a more conventional linear trend is found. The linear effect is significant, *b* = 0.513, *R-square* = 0.643, *p* < 0.001. The quadratic effect is also significant, *b* = 1.173, *R-square* = 0.784, *p* < 0.001, but this reflects the sharp increase at 15–20 h or more. There is no Goldilocks effect.

A similar comparison was made for the number of sites used for internet access, see Figure 3. The linear effect is significant, *b* = 0.448, *R-square* = 0.771, *p* < 0.001. The quadratic effect is also significant, *b* = 0.979, *R-square* = 0.796, *p* < 0.001. There is a curvilinear trend, with the least risk being at 2 places rather than 1 place, or 3 places or 4 + places. *T* tests showed that between 2 places and 1 place *t* = −0.089, 3 places *t* = −1.163, and 4+ places *t* = −0.673, none of these being significant. There is therefore a nonsignificant trend for a Goldilocks effect, of small effect size (Cohen’s *d* of 2 places with 1 place is 0.017, with 3 places is 0.090, with 4 places is 0.069).

Finally, comparison was made for number of activities on the internet, see Figure 4. There is a significant linear effect, *b* = 0.438, *R-square* = 0.704, *p* < 0.001. There is also a significant quadratic effect, *b* = 0.996, *R-square* = 0.794, *p* < 0.001, but this reflects the sharp increase in risk at 5–6 and 7+ activities. There is no Goldilocks effect.

### 3.3. Internet Use and Risk of Being a Frequent Cyber Victim

The following analyses only used the 163 students who had been cybervictims. A comparison of the risk of being a frequent cybervictim against how often a student uses the internet, is shown in Figure 5. The linear effect is significant, *b* = 0.739, *R-square* = 0.801, *p* ≤ 0.001; the quadratic effect is also significant, *b* = 1.819, *R-square* = 0.878, *p* < 0.001. There is a curvilinear trend, with the least risk being at several times a week rather than once a week/once a month, or than once a day or several times a day. However, *t* tests show that there are no significant differences between several times a week with once a week/once a month *t* = −1.360, or with once a day *t* = −1.535, or several times a day *t* = −1.902, all *n*-values. There is therefore a trend for a Goldilocks effect, of small effect size (Cohen’s *d* of several times a week with once a week/once a month is 0.345, with once a day is 0.352, with several times a day is 0.375).

**Figure 6:**
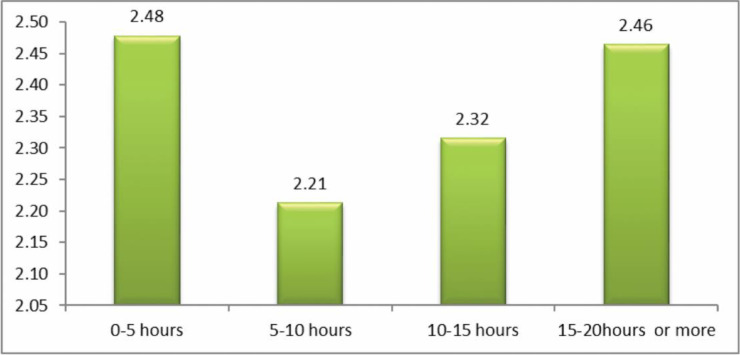
The risk of being a cybervictim (4-point scale), by time spent per week on the internet for the adolescents who were cyberbullied (*n* = 163).

A similar comparison for time spent per week on the internet is shown in Figure 6. The linear effect is significant, *b* = 0.898, *R-square* = 0.689, *p* ≤ 0.001. The quadratic effect is also significant, *b* = 2.244, *R-square* = 0.849, *p* ≤ 0.001. However, *t* tests show that there are no significant differences between 5–10 h and 0–5 h, *t* −1.858, and 10–15 h, *t*−0.595, and 15–20 or more hours, *t* = −1.409. There is therefore a trend for a Goldilocks effect, of small effect size (Cohen’s *d* between 5–10 h and 0–5 h is 0.369, with 10–15 h is 0.163, and with 15–20 or more hours is 0.313).

**Figure 7:**
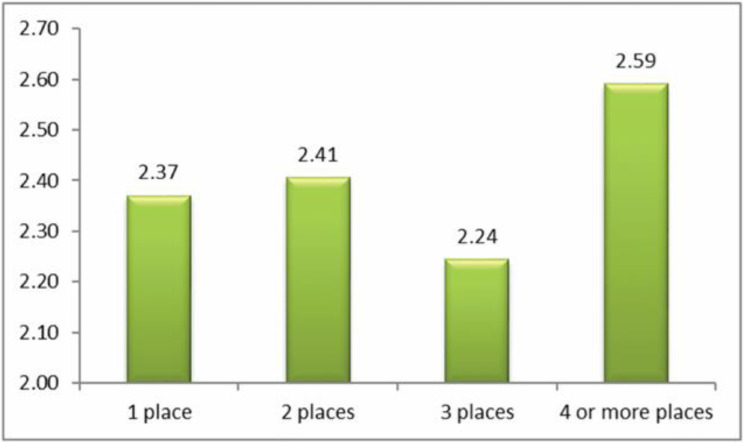
The risk of being a cybervictim (4-point scale), by numbers of sites used for internet for the adolescents who were cyberbullied (*n* = 163).

A further comparison was made for the number of sites used for internet access, see Figure 7. The linear effect is significant, *b* = 0.875, *R Square* = 0.790 *p* ≤ 0.001. The quadratic effect is also significant, *b* = 1.906, *R Square* = 0.874, *p* < 0.001. The lowest risk was for 3 places, compared to 1 or 2 places, or 4 or more places. However, *t* tests show that there are no significant differences between 3 places and 1 place, *t* = 0.697, and 2 places *t* = −1.202, and 4 or more places, *t* = −1.369. There is therefore a trend for a Goldilocks effect, of small effect size (Cohen’s *d* between 3 places with 1 place is 0.168, with 2 places is 0.246, and with 4 or more places is 0.388).

**Figure 8:**
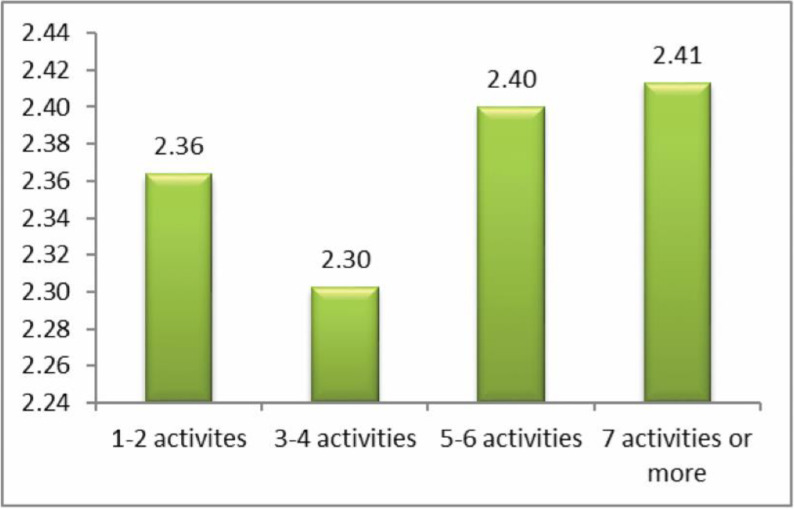
The risk of being a cybervictim (4-point scale), by number of activities on the internet for the adolescents who were cyberbullied (*n* = 163).

Finally, comparison was made for the number of activities on the internet, see Figure 8. The linear effect is significant, *b* = 0.775, *R Square* = 0.773, *p* ≤ 0.001. The quadratic effect is also significant, *b* = 1.953, *R Square* = 0.875, *p* ≤ 0.001. The lowest value is at 3–4 activities, compared to 1–2 activities, or 5–6 or 7 or more activities. However, *t* tests show that there are no significant differences between 3–4 activities compared to 1–2 activities, *t* = −0.349, 5–6 activities, *t* = −0.599, and 7 activities or more *t* = −0.661. There is therefore a trend for a Goldilocks effect, of very small effect size (Cohen’s *d* between 3–4 activities and 1–2 activities is 0.075, with 5–6 activities is 0.134, and with 7 activities or more is 0.139).

## 4. Discussion

This study provides new data on internet use in relation to risk of being a victim of cyberbullying, among adolescents in the southern provinces of Thailand. It provides some support for the Goldilocks hypothesis of risk, advanced by Przybylski and Weinstein [16].

As expected, the great majority of the adolescents in this sample had their own private mobile phones, and about half had smart phones. Their experience on the internet varied a lot in extent, but typically totaled several hours each week. Facebook was the most popular activity, in line with Pongput’s report [7]. Facebook is a popular activity for Thai adolescents, to communicate with friends conveniently on a 24-hour basis whether they were near or far away from them. We found that adolescents often used the internet to do homework or assignments from schools, and commonly used it to download songs, movies, and programs, to play games, to send and receive emails, to chat, to surf the internet, to use other social media such as MSN, Yahoo, and to use Skype (Table 1).

Although most internet activity can be fun or beneficial, there are also risks. One prominent risk is being a victim of cyberbullying. In this sample, about 14% of students could be characterized as cybervictims, over the past couple of months. We first examined the risk of being a cybervictim at all, in relation to four measures of intensity of internet use: how often it was used, how much time was spent on it per week, how many sites were used, and how many activities was it used for. The findings are shown in Figure 1, Figure 2, Figure 3 and Figure 4.

Figure 1 and Figure 3 give some support to the Goldilocks hypothesis. The lowest risk was not at the lowest level of internet use, but at a low/medium level of frequency of use, and of number of sites used for access. However, although the curvilinear trends are significant, the specific comparisons, with one exception for Figure 1, are not significant; and the effect sizes are very small. Figure 2 and Figure 4, although having significant curvilinear trends, do not support the Goldilocks hypothesis, and in fact show higher risks associated especially with spending very much time on the internet, in many types of activities.

We secondly examined the risk of being a frequent cybervictim, for those who had experienced a cyberattack. The findings, shown in Figure 5, Figure 6, Figure 7 and Figure 8, all have significant curvilinear effects overall, consistent with a Goldilocks effect. However, specific comparisons are not significant; and the effect sizes are small. The risk of becoming a frequent cybervictim (if victimized at all) tends to be moderate for using the internet only once a week or month, for 0–5 h/week, in 1 or 2 places, and for 1 or 2 activities. It tends to be less for using the internet several times a week, for 5–10 h/week, in 3 places, and for 3–4 activities. The highest risk tends to be for using the internet once or several times a day, 15–20 h/week or more, in 4 or more places, and for 5 or more activities. However, these analyses were under-powered in terms of sample size, so should be considered as indicative but requiring replication on larger samples.

In all cases, the highest risk is at the highest end of each scale. In fact, all eight comparisons (Figure 1, Figure 2, Figure 3, Figure 4, Figure 5, Figure 6, Figure 7 and Figure 8) have a significant linear effect. Those students who use the internet very intensively, are clearly at most risk. This is the finding of negative outcomes related to excessive internet use, reported extensively in the literature, and it is clearly true also for this sample of adolescents in Thailand.

However, for six of our eight comparisons, the significant curvilinear trend does suggest a Goldilocks effect, albeit of small or very small effect size. Those who only use the internet very infrequently or in limited ways, tend to be at increased risk. This finding from Thai adolescents is consistent with the data reported by Viner et al. [23] on an English sample of similar age, even though these latter authors did not explicitly mention or test the Goldilocks hypothesis. It is also consistent with the findings summarized by Twenge [21] on US and UK samples.

In our study, the Goldilocks finding is most consistent for the risk of being a frequent cybervictim, if you have been victimized at all. Why might this be so? Most adolescents in this sample used the internet several times a week, in several places, and for several purposes. It may be that those adolescents who only use the internet slightly, have not developed such good internet skills and are less able to cope with, or are more vulnerable to, possible cyberattacks. As with traditional bullying, those who appear vulnerable may be a tempting target for those who get gratification from bullying [42]. Low internet use may also be a sign of poorer competence generally, rather than specifically for the internet.

An alternative explanation, reversing the causal direction, would be to suppose that being a cybervictim leads one to avoid internet use. Avoidance strategies are fairly common in pupils who are bullied [43], and a study of coping strategies by cybervictims in Belgium found that they did tend to use passive coping strategies [44]. Data on coping strategies used, and especially longitudinal data, would be desirable to better consider this possibility. 

Besides its cross-sectional nature, other limitations of this study should be noted. One limitation was that the sample size was small, especially for some cybervictim frequency categories. In particular, the analyses with our subsample of 163 was under-powered given the Cohen’s *d* values that we obtained. Additionally, all the data are self-reported. Self-reported data is very commonly used in studies of internet use, and of bullying, and has the advantage that pupils know best about their own experiences. However, there is some danger of self-serving bias, for example being reluctant to admit to victimization. Peer nominations can be an excellent method for looking at associations with victim role, but are not so feasible for larger-scale studies. Additionally, being cybervictimised is an indirect measure of well-being, as we did not assess the extent of actual distress this caused. A final limitation, at least regarding contemporary relevance, is that data were gathered in 2013; usage patterns, and activities among adolescents are likely to have changed in the intervening years.

## 5. Conclusions

This study assessed four measures of extent of internet use against one important measure of well-being, namely being a victim of cyberbullying. Some support was found for the generally assumed linear displacement hypothesis, in that the highest risks were associated with the most intensive internet use. However, some findings also provided support for the curvilinear Goldilocks Hypothesis. This was especially so as regards the risk of being a frequent cybervictim, once having experienced a cyberattack. One possible explanation for this may be lower coping skills in those who use the internet rather infrequently. However, these latter findings are qualified by small sample size. At a practical level, a provisional recommendation for lowering risks of being cyberbullied would be for parents not to unduly shelter their adolescent child from internet experience, but to be concerned or take preventative action if the internet is being used several times a day, 15–20 h/week or more, in 4 or more places, and for 5 or more activities. Future research examining risks of internet use should go beyond linear correlations, to provide a fuller assessment of how measures of internet use relate to measures of well-being, following the example of Przybylski and Weinstein [16]. In addition, future research can examine the reasons for any curvilinear trends obtained, and in particular the reasons for the extent of increased risk related to the lowest levels of internet use. Such findings will be of practical significance. If substantiated on larger samples, a curvilinear relationship between internet use and cyberbullying would suggest a ‘safe zone’ for adolescent internet use, bringing its benefits while minimizing risks of cyberbullying. It would help in giving a sound empirical base for considering when frequent internet use becomes ‘excessive’, while acknowledging the benefits of becoming familiar with the risks of the internet and developing coping strategies; an important aspect of e-safety education.

## Figures and Tables

**Table 1:** Numbers and percentages for scores of internet use (total *n* = 1140).

**How Often do You Use the Internet?**	***n***	**(%)**
Once a month	31	2.7
Once a week	179	15.7
Several times a week	321	28.2
Once a day	265	23.2
Several times a day	344	30.2
**How long do you spend on the internet per week?**		
0–5 h	594	52.2
5–10 h	297	26.0
10–15 h	118	10.4
15–20 h	62	5.4
20 or more hours	68	6.0
**Where are you most likely to use the internet?**		
In my bedroom	177	15.5
At home, not my bedroom	743	65.2
At school	429	37.6
Friend’s house	200	17.5
At work	36	3.2
At the local library	63	5.5
Internet café	810	71.1
At a relative’s house	218	19.1
Other places not above	2	0.1
**What activities do you use the internet for?**		
Surfing the Net	381	33.4
Chat rooms	407	35.7
Send/receive emails	386	33.9
Schoolwork	862	75.6
Downloading music, films or programs	671	58.9
Playing games	629	55.2
Online shopping	95	8.3
Facebook	879	77.1
Skype	83	7.8
Other social networking sites	296	26.0
